# Comparative study of a broad qPCR panel and centrifugal flotation for detection of gastrointestinal parasites in fecal samples from dogs and cats in the United States

**DOI:** 10.1186/s13071-023-05904-z

**Published:** 2023-08-16

**Authors:** Christian M. Leutenegger, Cecilia E. Lozoya, Jeffrey Tereski, Jan Andrews, Kelly D. Mitchell, Cathy Meeks, Jennifer L. Willcox, Gregory Freeman, Holly L. Richmond, Christian Savard, Michelle D. Evason

**Affiliations:** 1 Antech Diagnostics, Inc, Fountain Valley, CA USA; 2 BioVet Inc, Saint-Hyacinthe, QC Canada

**Keywords:** PCR, Gastrointestinal, Parasites, Resistance, *Giardia*, Zoonotic, *Ancylostoma*

## Abstract

**Background:**

For decades, zinc sulfate centrifugal fecal flotation microscopy (ZCF) has been the mainstay technique for gastrointestinal (GI) parasite screening at veterinary clinics and laboratories. Elsewhere, PCR has replaced microscopy because of generally increased sensitivity and detection capabilities; however, until recently it has been unavailable commercially. Therefore, the primary aim of this study was to compare the performance of real-time PCR (qPCR) and ZCF for fecal parasite screening. Secondary aims included further characterization of markers for hookworm treatment resistance and *Giardia* spp. assemblages with zoonotic potential and qPCR optimization.

**Methods:**

A convenience sampling of 931 canine/feline fecal samples submitted to a veterinary reference laboratory for routine ZCF from the Northeast US (11/2022) was subsequently evaluated by a broad qPCR panel following retention release. Detection frequency and agreement (kappa statistics) were evaluated between ZCF and qPCR for seven GI parasites [hookworm/(*Ancylostoma* spp.), roundworm/(*Toxocara* spp.), whipworm/(*Trichuris* spp.), *Giardia* *duodenalis*, *Cystoisospora* spp., *Toxoplasma* *gondii*, and *Tritrichomonas* *blagburni*] and detections per sample. Total detection frequencies were compared using a paired *t*-test; positive sample and co-infection frequencies were compared using Pearson’s chi-squared test (*p* ≤ 0.05 significant) and qPCR frequency for hookworm benzimidazole (BZ) resistance (F167Y) and zoonotic *Giardia* spp. assemblage markers calculated. Confirmatory testing, characterization, and qPCR optimization were carried out with Sanger sequencing.

**Results:**

qPCR detected a significantly higher overall parasite frequency (*n* = 679) compared to ZCF (*n* = 437) [*p* =  < 0.0001, *t* = 14.38, degrees-of-freedom (*df*) = 930] and 2.6 × the co-infections [qPCR (*n* = 172) vs. ZCF (*n* = 66)], which was also significant (*p* =  < 0.0001, *X*^*2*^ = 279.49; *df* = 1). While overall agreement of parasite detection was substantial [kappa = 0.74; (0.69–0.78], ZCF-undetected parasites reduced agreement for individual and co-infected samples. qPCR detected markers for *Ancylostoma* *caninum* BZ resistance (*n* = 5, 16.1%) and *Giardia* with zoonotic potential (*n* = 22, 9.1%) as well as two parasites undetected by ZCF (*T.* *gondii*/*T.* *blagburni*). Sanger sequencing detected novel roundworm species, and qPCR optimization provided detection beyond ZCF.

**Conclusions:**

These results demonstrate the statistically significant detection frequency advantage offered by qPCR compared to routine ZCF for both single and co-infections. While overall agreement was excellent, this rapid, commercially available qPCR panel offers benefits beyond ZCF with detection of markers for *Giardia* assemblages with zoonotic potential and hookworm (*A.* *caninum*) BZ resistance.

**Graphical Abstract:**

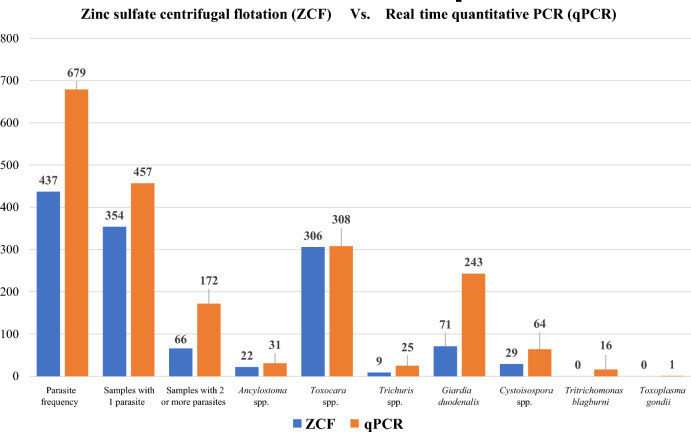

## Background

Gastrointestinal (GI) parasitism is common in domestic dogs and cats in North America and can cause significant clinical disease, particularly in puppies and kittens [1]. In both human and veterinary medicine, diagnosis of (and routine screening for) GI parasites has been performed through centrifugal flotation and microscopic ova and parasite (O&P) identification for more than a century [2]. While fecal centrifugal flotation using zinc sulfate (ZCF) remains standard in many veterinary hospitals, as well as reference laboratories, it has limitations in sensitivity, is operator expertise dependent, and can only detect what can be visualized microscopically [3]. Morphometric inconsistencies in egg and cyst sizes for particular parasites and the presence of artifacts mimicking parasite structures are additional factors contributing to misidentification of GI parasites with this method [4]. Moreover, routine ZCF is unable to delineate subsets of parasites with zoonotic potential or anthelmintic resistance associated markers. The traditional advantage of ZCF for veterinary clinical use has been affordability. This feature also makes ZCF suitable as a high-throughput test method in reference laboratories.

With the ascent of functional genomic tools and the generation of abundant parasite gene sequence information, the rapidly expanding field of molecular diagnostics has provided a novel opportunity for GI parasite fecal detection [5]. Targeting DNA allows highly specific detection of clinically relevant parasite strains, as DNA is present in any cell of the organism, including the epithelial cells shed from a worm. A unique sensitivity enhancement is also possible by targeting both parasitic DNA and RNA. Reverse transcription real-time quantitative polymerase chain reaction (RT-qPCR) further enhances analytical detection and diagnostic sensitivity, while allowing retention of accurate quantitative assessments of parasite burden [6–8]. This has been shown in veterinary studies reporting higher sensitivity for coproPCR as compared to ZCF for certain GI parasites [9–11].

As such, the availability of parasite genetic information combined with molecular testing capabilities could propel this innovation to become the new reference method for veterinary diagnostic testing and fecal GI parasite surveillance. Real-time polymerase chain reaction (qPCR) has been shown to be safe to use in reference laboratories and provides the possibility of commercial automation and high throughput reporting turnaround times (TAT). Importantly, molecular diagnostics offer a number of distinct clinical advantages, such as maximizing the recovery of inhibition-free PCR amplifiable DNA and RNA. For example, research has shown that amplifiable hookworm DNA can be recovered from stool samples stored at room temperature for up to 10 days without preservative and 40 days when refrigerated [12]. This stability of parasite DNA enables flexibility and practicality for pet owners. Less time and temperature sensitive samples allow for improved convenience in fecal ‘drop-off’ or shipment to the veterinary clinic and reference laboratory without sacrificing the quality and reliability of test results.

Further benefits of molecular diagnostics in routine dog and cat GI parasite screening include their ability to detect genetic markers which confer resistance against anthelmintic drugs, i.e. hookworm benzimidazole (BZ) treatment resistance [13] and *Giardia* with potential zoonotic assemblages [14], and allow for *One* *Health* parasite surveillance information [15]. Additonal PCR advantages are the method’s rapid adapatability and innovation potential, which can lead to identification of novel resistance markers, such as the F167Y genetic marker for BZ hookworm treatment resistance [16], and parasite strains, like the differentiation of four previously unidentifiable *Ancylostoma* strains [17]. This information can provide clinical treatment information and antimicrobial therapy guidance, thus promoting actionable antimicrobial stewardship.

The current guidelines from the Companion Animal Parasite Council (CAPC), Canadian Parasitology Expert Panel (CPEP), and European Scientific Counsel for Companion Animal Parasites (ESSCAP) for endoparasite prevention in dogs and cats advise regular routine screening for GI parasites. However, despite the myriad advantages offered by molecular-based fecal diagnostic testing (qPCR), this method has been infrequently employed for routine veterinary wellness. This has been primarily due to limited qPCR availability (i.e. only at research facilities), high cost, and slow TAT, which has restricted practical clinical use. In answer to these expert- and evidence-based recommendations for routine veterinary GI parasite screening and surveillance, and due to the increasing need for a test methodology capable of detecting growing *One* *Health* concerns (e.g. anthelmintic resistance, zoonotic risk), a rapid, broad-spectrum qPCR GI parasite panel has become available in the reference laboratory setting [13, 15].

Regardless of the many overt advantages of molecular diagnostics over traditional centrifugal flotation (ZCF), there is always a need for comparative studies based in field settings and commercial accountability towards quality assessment, innovation, and efficiency, particularly as novel parasites and markers emerge. The primary aim of our field validation study was to evaluate the performance of this qPCR panel compared to standard zinc sulfate centrifugal flotation and determine the level of agreement for fecal GI parasite screening for seven common parasites in a collection of samples from privately owned dogs and cats in the USA. Additional aims were to describe coinfection detection frequency, frequency of detection of markers for hookworm BZ resistance and zoonotic *Giardia* spp. assemblages and utilize elucidated roundworm species for qPCR assay optimization in this population.

## Methods

### Sample collection and nucleic acid extraction

A convenience sampling of archival canine and feline fecal samples (*n* = 931; canine = 645; feline = 266; not reported = 20) originally submitted to a large veterinary reference laboratory for routine ZCF from the Northeast USA in November 2022 was selected for further evaluation. ZCF (specific gravity of 1.18 ± 0.005) was processed (using 2 g fecal material) and results provided as per standard operating procedure for this laboratory at the time of submission; what remained of each sample was refrigerated at 4 ºC until expiration of the 7-day hold period. Following retention release, these samples underwent total nucleic acid extraction with optimized protocols [13]. Between 150 to 250 mg of fecal material was incubated in a guanidinium-based lysis solution and mechanically homogenized using pre-loaded bead vials (Spex SamplePrep, Metuchen, NJ), and total nucleic acid was extracted on a KingFisher Apex (13, Thermo Fisher, Waltham, MA, USA).

### PCR designs and validation

Real-time quantitative PCR assays were designed using sequences deposited in GenBank (Table 1). The 24 qPCR test parasite panel is a proprietary diagnostic test offered commercially (KeyScreen® GI Parasite PCR, Antech Diagnostics, Inc.). Previously described assay designs and validation protocols were followed for rigorous analytical and clinical validation [18]. The parasite qPCR panel includes a quantitative internal sample control (ISC) consisting of a pan-bacterial qPCR test based on 16S ribosomal RNA gene sequences and a spike-in internal positive control (IPC) to control for remaining PCR inhibitors [19]. The remaining 22 qPCR consists of 20 parasite-specific tests and genetic markers detecting the F167Y benzimidazole resistance in *Ancylostoma* *caninum* and *Giardia* strains with potentially zoonotic assemblages A and/or B [13, 19, 20]. The specificity definitions and target genes for the seven parasite qPCR tests used to compared with ZCF protocols in this work are shown in Table 1. Real-time PCR reactions were carried out in a LC480 (Roche Molecular Diagnostics, Indianapolis, IN) using standard protocols [13].

**Table 1:** Target genes, GenBank accession numbers, and specificity definitions of the seven parasite qPCR used to compare with zinc sulfate centrifugal flotation protocols

Parasite	Target gene	GenBank accession no.	Specificity definitions
*Ancylostoma* spp.	ITS-1	KC755026	*A.* *caninum,* *A.* *tubaeforme,* *A.* *braziliense,* *A.* *ceylanicum,* *A.* *duodenalis*
*Toxocara* spp.	5.8S	JF837169	*T.* *canis,* *T.* *cati,* *T.* *leonina,* *Baylisascaris* *procyonis*
*Trichuris* spp.	ITS-1	GQ352558	*T.* *vulpis,* *T.* *sagitana*
*Giardia* *duodenalis*	ssrRNA	MF163432	*G.* *duodenalis*
*Cystoisospora* spp.	ssrRNA	KT184368	*C.* *canis,* *C.* *ohioensis*
*Tritrichomonas* *blagburni*	ssrRNA	AF466749	*T.* *blagburni* *(*formerly *T.* *foetus)*
*Toxoplasma* *gondii*	ITS-1	KP895872	*T.* *gondii*

### Confirmatory testing and qPCR optimization

Discordant *Toxocara* spp. sample results (defined as those samples detected for *Toxocara* species by ZCF and initially undetected by qPCR or initially detected by qPCR and undetected by ZCF) were further characterized by Sanger sequencing protocols as previously described [13]. These data were then utilized for qPCR test performance optimization for the instances where qPCR was initially undetected for *Toxocara* spp. Confirmatory testing for *Ancylostoma* spp., hookworm BZ resistance marker, and/or marker *Giardia* *duodenalis* assemblage A/B with potential for zoonosis was also conducted by Sanger sequencing [13].

### Data analyses

Results from the ZCF and qPCR methologies were evaluated to determine frequency of detection for seven common canine and feline GI parasites: hookworm (*Ancylostoma* spp.), roundworm (*Toxocara* spp.), whipworm (*Trichuris* spp.), *G.* *duodenalis*, *Cystoisospora* spp., *Tritrichomonas* *blagburni*, and *Toxoplasma* *gondii*. Frequency calculations included: overall parasite detection (defined as the total number of parasites detected by each method), parasite-detected samples (defined as the total number of samples with at least one parasite detected), and co-infection (defined as two or more parasites detected for an individual sample), and levels of co-infection were further subclassified by the number of parasites detected for each co-infected sample. Overall difference in frequency of parasites detected by each method was evaluated using a paired *t*-test. A Pearson’s chi-square test was used to compare the frequency of parasite-detected samples and samples with co-infection detected between the two methodologies. This was reported as the chi-square value, degrees of freedom (*df*), and a corresponding *p*-value, where < 0.05 was considered significant. Kappa statistics were used to determine the level of agreement between the molecular qPCR test and the ZCF method where the interpretation would be as follows: values ≤ 0 as indicating no agreement, 0.01–0.20 as none to slight, 0.21–0.40 as fair, 0.41–0.60 as moderate, 0.61–0.80 as substantial, and 0.81–1.00 as almost perfect agreement [21]. Agreement was evaluated for the number of parasite-detected samples, samples with co-infection detected, and each parasite; this was reported as overall, positive, and negative agreements with their corresponding Wald *Z* 95% confidence intervals (CI). Frequencies were also calculated for qPCR-detected hookworm benzimidazole (BZ) resistance (F167Y) and zoonotic *Giardia* spp. assemblage (A and/or B) markers. Statistics were calculated using commercially available software [Analyse-it for Microsoft Excel (version 2.30), Leeds, UK].

## Results

### Overall parasite detection by ZCF and qPCR

For the seven common canine and feline parasites included in this study, qPCR detected a 1.6 × greater overall frequency of parasites (*n* = 679) compared to ZCF (*n* = 437), and this difference was statistically significant (*p* =  < 0.0001, *t* = 14.39, *df* = 930). When evaluating the frequency of parasite-detected samples, qPCR detected a statistically significantly greater frequency (*n* = 457) compared to ZCF (*n* = 354) (*p* =  < 0.0001, *X*^*2*^ = 528.52, *df* = 1) (Table 2).

**Table 2:** Comparison of the number of canine and feline fecal samples with at least one of the seven evaluated parasite species/genus detected and those with co-infection(s) for zinc sulfate centrifugal flotation (ZCF) and the qPCR panel

	ZCF	qPCR	Pearson’s chi-square	Degrees of freedom	*P*-value
Samples with at least 1 parasite species/genus detected	354	457	528.52	1	< 0.0001
Co-infection, samples with 2 or more detected	66	172	279.49	1	< 0.0001
Co-infection, 2 parasites	51	131	152.60	1	< 0.0001
Co-infection, 3 parasites	13	33	207.63	1	< 0.0001
Co-infection, 4 parasites	2	7	264.57	1	< 0.0001

### Detection comparison for each of the seven parasites by qPCR and ZCF

**Fig. 1:**
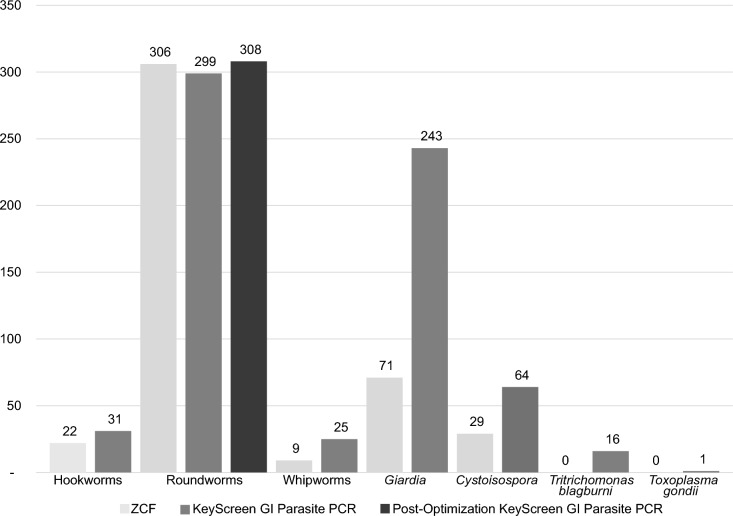
Method comparison overview of zinc sulfate centrifugation flotation (ZCF) and qPCR frequency detection (*n*) for hook-, round-, and whipworms, along with *Giardia*, *Cystoisospora* spp., *Tritrichomonas* *blagburni*, and *Toxoplasma* *gondii*. The roundworm totals have an additional bar corresponding to the results of the optimized qPCR test

Of the seven individual parasites compared, more GI parasites were detected by the qPCR test than by fecal ZCF for six of the seven parasites evaluated (Fig. 1 and Table 3). Detection frequency for qPCR as compared to ZCF (Table 2) was increased by 1.4 times for hookworms (*Ancylostoma* spp.), 3.4 times for *G.* *duodenalis*, 2.2 times for *Cystoisospora* spp., and 2.8 times for whipworms (*Trichuris* spp.), respectively. No *T.* *blagburni* or *T.* *gondii* was found by ZCF but 16 and one sample, respectively, were detected by qPCR. For roundworms, ZCF detected 1.02 times more *Toxocara* spp. than qPCR, with detection of 306 (ZCF) compared to 299 by qPCR (Table 4).

**Table 3:** Overview of zinc sulfate centrifugal flotation (ZCF) and qPCR parasite detection frequencies, co-infection frequencies, and agreement (overall, positive, and negative)

	ZCF *n* (frequency %)	qPCR *n* (frequency %)	Kappa (Wald Z 95% CI)	Positive agreement (95% CI)	Negative agreement (95% CI)
Samples with at least 1 parasite detected	354 (38%)	457 (49%)	0.74 (0.69–0.78)	0.97 (0.95–0.99)	0.8 (0.77–0.83)
All co-infections, 2 or more parasites detected	66 (7.1%)	172 (18.5%)	0.48 (0.4–0.55)	0.96 (0.88–0.98)	0.87 (0.85–0.89)
Co-infection, 2 parasites detected	51 (5.5%)	131 (14.1%)	0.36 (0.27–0.44)	0.725 (0.59–0.83)	0.89 (0.87–0.91)
Co-infection, 3 parasites detected	13 (1.4%)	33 (3.5%)	0.423 (0.24–0.6)	0.77 (0.5–0.92)	0.98 (0.96–0.98)
Co-infection, 4 parasites detected	2 (0.2%)	7 (0.7%)	0.44 (0.04–0.85)	1.0 (0.34–1)	0.99 (0.98–0.99
Co-infection, 5 parasites detected	0 (0%)	1 (0.1%)	N/A	N/A	0.99 (0.99–1.0)
*Ancylostoma* spp.	22 (2.4%)	31 (3.3%)	0.79 (0.66–0.91)	0.96 (0.78–0.99)	0.99 (0.98–0.99)
*Toxocara* spp.	306 (32.9%)	299 (32.1%)	0.94 (0.92–0.96)	0.95 (0.92–0.97)	0.99 (0.97–0.99)
*Trichuris* spp.	9 (1%)	25 (2.7%)	0.52 (0.32–0.73)	1 (0.7–1)	0.98 (0.97–0.99)
*Giardia* *duodenalis*	71 (7.6%)	243 (26.1%)	0.36 (0.3–0.42)	0.97 (0.9–0.99)	0.8 (0.77–0.82)
*Cystoisospora* spp.	29 (3.1%)	64 (6.9%)	0.58 (0.47–0.7)	0.97 (0.83–0.99)	0.96 (0.95–0.97)
*Tritrichomonas* *blagburni*	– (0%)	16 (1.7%)	N/A	N/A	0.98 (0.97–0.99)
*Toxoplasma* *gondii*	– (0%)	1 (0.1%)	N/A	N/A	0.99 (0.99–1)

*CI*  confidence interval

**Table 4:** The 2 × 2 chart for *Toxocara* spp. zinc sulfate centrifugation flotation (ZCF) and qPCR results

A	qPCR		Totals
	Positive	Negative	
ZCF			
Positive	290	16	**306**
Negative	9	616	**625**
Totals	**299**	**632**	**931**

B	qPCR		Totals
	Positive	Negative	
ZCF			
Positive	299	7	**306**
Negative	9	616	**625**
Totals	**308**	**623**	**931**

A: Original performance data. B: Performance data after *Toxocara* spp. PCR optimization with newly characterized feline roundworm species

### Co-infections detected by qPCR and ZCF

**Fig. 2:**
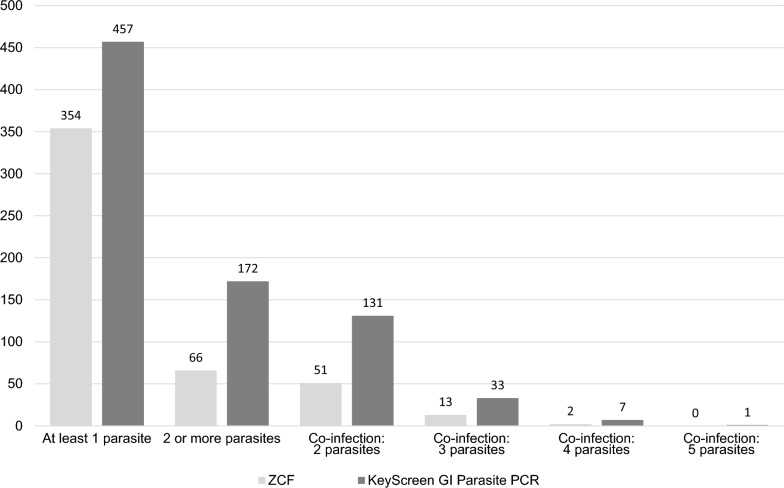
Frequency comparison of zinc sulfate centrifugation flotation (ZCF) and qPCR for total samples with at least one parasite detected, co-infection detected samples (two or more), and corresponding frequencies for the levels of co-infection (two parasites, three parasites, four parasites, and five parasites)

Co-infections, where at least two parasites were detected, were identified by qPCR in 172 samples as compared to 66 parasite-detected samples for ZCF. When compared, these frequencies were significantly different (*X*^*2*^ = 279.49, *df* = 1, and *p* =  < 0.0001) (Table 2). Of these co-infections, qPCR detected up to five parasite species in a single sample and up to four for ZCF (Table 3, Fig. 2). At all levels of co-infection (two to five parasites detected in a single sample), qPCR detected a greater frequency compared to ZCF, and this difference in frequency was statistically significant (Table 2, Fig. 2).

### Agreement for parasite detection between ZCF and qPCR

Next, frequencies of overall parasite detection, parasite-detected samples, each of the seven individual parasites, and samples with co-infections for ZCF and qPCR were analyzed for agreement between the two methodologies (Table 3). For the overall frequency of parasite-detected samples, agreement was considered substantial with kappa = 0.74 (95% CI 0.69–0.78). When evaluating the agreement between the individual parasites, near perfect agreement was found for *Toxocara* spp. and substantial agreement for *Ancylostoma* spp. As expected, for parasites where qPCR detection frequency far surpassed that of ZCF (*G.* *duodenalis*, *Trichuris* spp., *Cystoisospora* spp., *T.* *blagburni*, and *T.* *gondii*), agreement was only fair to slight (Table 3). Similarly, the differences in frequency of detection for these parasites also impacted the agreement between ZCF and qPCR for co-infections, which was fair [kappa 0.48 (0.4–0.55)] overall.

### Roundworm confirmatory testing and *Toxocara* spp. qPCR performance optimization

To identify discordant *Toxocara* spp. cases for confirmatory testing, the above data were further analyzed in 2 × 2 chart analyses (Table 4). A total of 25 samples were identified as discordant for roundworm detection between the two methodologies with nine being qPCR detected and undetected by ZCF and 16 being detected by ZCF but undetected by qPCR. When further characterized by Sanger sequencing, the nine ZCF undetected samples were confirmed to be *Toxocara* spp. For the 16 ZCF-detected/qPCR-undetected roundworm samples, Sanger sequencing targeted three different roundworm genes (16S ribosomal RNA, ITS-2, and the mitochondrial gene COX-2), and nine of 16 were confirmed to be true-positive feline roundworm samples. Sequence analysis on the ITS-2 gene and comparison to the primer and hydrolysis probe used to identify the roundworm at the species level revealed several previously unknown sequence variations in the feline roundworm *Toxocara* *cati*. When these sequence variations were incorporated into the qPCR assay, the test detected all nine samples with a novel feline roundworm species. The remaining roundworm samples were found to be of canine origin with novel genotypes. Sequencing workup and updating of the *Toxocara* spp. roundworm qPCR are ongoing.

### Genetic markers for anthelmintic resistance in hookworm and zoonotic potential in *Giardia duodenalis*

The genetic marker F167Y indicating BZ resistance was detected in five of the 31 *Ancylostoma* spp.-detected samples (16.1%). All *Ancylostoma* spp. (*n* = 31) were detected in canine submissions and were sequence confirmed to be *A.* *caninum.* In addition, the five samples with the F167Y genetic marker in *A.* *caninum* were confirmed to contain the 167Y mutation by variant calling of Sanger sequencing trace chromatograms as described [13].

*Giardia* spp. was detected in 243 of 931 samples (26.1%); of those, 22 (9.1%) had detection of the *G.* *duodenalis* marker for zoonotic potential. The *G.* *duodenalis* assemblage A and B determination was performed using beta-giardin specific locked nucleic acid containing hydrolysis probes. The *G.* *duodenalis* assemblage A/B-detected samples were confirmed by Sanger sequencing.

## Discussion

To the authors’ knowledge, this represents the first study to compare the performance of ZCF to this commercially available broad-spectrum qPCR panel for intestinal parasite identification in a collection of fecal samples from privately owned dogs and cats. When comparing the overall performance to detect parasite load, frequency of parasite-detected samples, and ability to detect co-infection, qPCR detected a statistically significant greater frequency compared to ZCF. This difference was especially striking when evaluating the number of co-infections, where qPCR identified nearly three times as many. Agreement calculations demonstrate the overall ability to detect parasite burden and parasite-detected samples between ZCF and qPCR to be substantial however also highlights the specific parasites where ZCF had a reduced to absent ability to detect (*G.* *duodenalis*, *Trichuris* spp., *Cystoisospora* spp., *T.* *blagburni*, and *T.* *gondii)* where agreement was only slight to fair. These data suggest that while ZCF remains a highly affordable, fast, and widely available GI parasite screening test, molecular diagnostics such as this qPCR panel are poised to undergo consideration as a reference method for parasite detection because of its ability to maintain affordability and rapid turnaround while additioanlly providing the advantage of increased parasite detection.

While commonly used by many veterinary clinics and reference laboratories, the limitations of ZCF for intestinal parasite screening have been demonstrated previously. This method is outperformed by the double centrifugation method and fails to identify smaller parasite structures such as whipworm eggs because of the relatively low density of zinc sulfate [22]. This may be one explanation for ZCF’s inability to detect certain parasites compared to qPCR in this study and contributed to a reduction in agreement between the two methods as well as co-infection. However, double centrifugation methods are less time and cost efficient and therefore limit the accessibility of this test. Molecular diagnostics on the other hand have been previously described to provide an advantage in diagnostic sensitivity in multiple applications.

The ‘omics’ revolution in the field of biological sciences over the past 2 decades has vastly improved access to molecular and genetic resources of parasites. This explosion of research and collaboration has opened doors for the deployment of molecular diagnostic techniques with improved performance [5]. Additionally, advanced molecular tools such as CRISPR technologies are furthering the functional understanding of new mutations correlating with anthelmintic resistance [23]. These molecular techniques have the unique ability to increase sensitivity and specificity of diagnostic tools, enabling the generation of fecal surveillance data, which supports developing new control initiatives for parasitic diseases and alerts us to GI parasites of *One* *Health* concern, e.g. *Giardia* strains with zoonotic potential and *A.* *caninum* treatment-resistant hookworms [13, 15].

There is an increasing body of evidence supporting the superiority of molecular tests to detect parasitic infections with higher sensitivity, more breadth, and more functional qualities such as anthelmintic resistance and zoonotic variations [9, 23–29]. Conventional fecal diagnostic methods, like centrifugal flotation and antigen detection, are solely based on morphological features or secretory/excretory antigens [30]. These methods also lack the ability to read into the functional differences of parasites or detect antimicrobial treatment resistance mechanisms or differences in parasite zoonotic or non-zoonotic potential. In addition, the morphological assessment of parasitic eggs or cysts, in particular for *Giardia*, often cannot be identified or differentiated by ZCF in a general practitioner setting [31]. Similarly, biological structures such as pollen grains, plant and yeast cells, or fungal elements may be misidentified as parasitic structures by fecal centrifugal flotation techniques [32], while variations in egg size due to sample age, temperature, humidity, and even anthelmintic therapy can make accurate fecal microscopy more difficult [33, 34]. These factors can lead to a relative lack of specificity, which may impact the overall sensitivity of fecal ZCF.

In molecular diagnostics, Sanger sequencing can be used to confirm the presence or absence of parasites when conventional tests are negative. This provides an agile platform for sample characterization and subsequent assay optimization. In our study, these protocols were implemented to confirm discordant roundworm-detected samples, hookworm resistance marker-detected samples, as well as the *Giardia* strains with zoonotic potential. Further confirmatory testing was performed for a selection of hookworm samples, 12 whipworm samples, and two of the 36 *Cystoisospora* samples. Additionally, of the 134 qPCR-detected *Giardia* *duodenalis* samples which were ZCF negative, we selected six samples, all of which were confirmed by qPCR as *G.* *duodenalis*. Based on the unique ability to prove the existence of nucleic acid for particular parasites by Sanger sequencing using outside primers, the specificity of molecular tests can be adjusted in most qPCR applications to approach 99%, as described in our work.

Fecal qPCR, like all biological tests, is not perfect, and potential for false positives and negatives remains. In our study we observed a low frequency of discordant results, some of which represented ZCF-positive but qPCR-undetected samples. The example in this study relates to those roundworm samples where *Toxocara* was identified by ZCF but undetected by qPCR. One explanation relates to the potential subjectivity of microscopy and its potential for misidentification of plant, yeast, or fungal material as GI parasite structures. However, another explanation, and as shown with our novel roundworm species findings, morphologically clearly identifiable *Toxocara* eggs can be observed with fecal ZCF but are reported undetected by molecular methods. As in this study, the phenomenon can be secondary to the existence of novel parasite genotypes which are not detected by fecal qPCR because of mismatches on the primers and/or probes of a given qPCR test. Characterization of new genotypes, therefore, is of great use in molecular diagnostics to minimize false-negative results and further elevate molecular diagnostics to the reference method status. In our study, nine of the 16 ZCF-positive roundworms were confirmed as *T.* *cati* with unique sequence variations rendering the qPCR test undetected. Updating the qPCR primers, and if necessary hydrolysis probe sequences, to include those novel strains into the roundworm specificity definition is a necessary and elegant way to expand the performance of the *Toxocara* spp. qPCR. Existence of distinct species-adapted genotypes as a result of biological adaption to their respective host has been described for other parasites, such as the separation of the tapeworm *Dipylidium* *caninum* into a feline and a canine genotype [35].

Limitations of this study include those inherent to retrospective study design including a lack of clinical data regarding presenting signs, medication history, reason for submission, etc. Given the time of year and region where the samples were collected, it is likely that the parasite detection frequency results should not be extrapolated or interpreted as representative of prevalence as that was outside the scope of this study. For example, the age, region, and season could have biased this population toward a greater number of roundworm infections as has been described previously in North America [1, 36]. Another limitation of this study is the lack of corresponding double-centrifugation results for this collection of samples to provide a reference method for comparison. While ideal, double centrifugation is not representative of intestinal parasite screening for veterinary patients from a commercial laboratory, and the authors of this study opted instead to compare two tests readily available to most clinicians. Future studies would be necessary to compare qPCR and double centrifugation using different specific gravities of sugar and salt solutions to determine their diagnostic concordance.

As mentioned above, roundworms comprised the largest proportion of parasite-detected samples. This provided the opportunity to investigate the presence of previously unknown roundworm nucleotide regions and variants. Diligence in the selection of nucleotide regions for the design of qPCR tests is of upmost importance. This is shown in our work through the roundworm example, where unknown genotypes or subtypes of particular nematode species can impact the sensitivity of molecular methods. However, due to molecular advancements in identifying previously unknown nucleotide sequences through conventional Sanger sequencing or any of the newer deep sequencing technologies, qPCR tests can be evolved and improved to detect novel sequence variations. As evidenced with the updated roundworm qPCR test, a diagnostic sensitivity of > 99% can be achieved when sequence mismatches on primers and probes are eliminated. This allows the detection of strains of clinical veterinary relevance with the highest analytical and diagnostic accuracy. Furthermore, using outside sequencing primers can not only discover new parasite genotypes but also confirm discrepant positive qPCR results. Additionally, the species-specific identification of *T.* *cati* in dogs likely indicates GI parasite passage, rather than true infection, and type of finding provides valuable information for the practitioner. Taken together, new genetic information can be used to further innovate and fine-tune molecular tests to detect previously undetected genotypes and is a key aspect of bringing molecular testing to the forefront of endoparasite surveillance and wellness screening in veterinary medicine.

## Conclusions

Molecular diagnostics using fecal qPCR testing are poised to claim reference method status due to their superior diagnostic performance in detection of GI parasites. Centrifugal fecal flotation methods can provide high-quaility, affordable, and fast results, particularly when performed by highly trained personnel in a reference laboratory setting. However, ZCF is limited in ability to detect parastites with unstable or difficult to recognize morphological structures. Furthermore, fecal microscopy cannot determine the presence of anthelmintic drug resistance or the presence of particular zoonotic markers. The diagnostic performance calculations described in our work provide evidence to support that molecular tests for GI parasites have superior performance compared to conventional ZCF. Affordable, rapid, and commercially available molecular methods (qPCR) of fecal GI parasite detection have the potential to improve veterinary patient care and outcomes and offer the possibility to become the tests of choice for GI parasite wellness screening and *One* *Health* surveillance and to follow antimicrobial stewardship guidelines.

## Data Availability

The dataset used and analyzed during this study are available from the corresponding author on reasonable request.

## Abbreviations

ZCF: Zinc sulfate centrifugal flotation
GI: Gastrointestinal
qPCR: Real-time quantitative PCR
BZ: Benzimidazole
TAT: Turnaround time

## References

[1] Drake J, Carey T (2019). Seasonality and changing prevalence of common canine gastrointestinal nematodes in the USA. Parasit Vectors.

[2] Telemann W (1908). Eine Methode zur Erleichterung der Auffindung von Parasiteneiern in den Faeces. Dtsch Med Wochenschr.

[3] Faust EC, Sawitz W, Tobie J, Odom V, Peres C, Lincicome DR (1939). Comparative efficiency of various technics for the diagnosis of protozoa and helminths in feces. J Parasitol.

[4] Colmer-Hamood JA (2001). Fecal microscopy artifacts mimicking ova and parasites. Lab Med.

[5] McVeigh P (2020). Post-genomic progress in helminth parasitology. Parasitology.

[6] Hodgson SH, Douglas AD, Edwards NJ, Kimani D, Elias SC, Chang M (2015). Increased sample volume and use of quantitative reverse-transcription PCR can improve prediction of liver-to-blood inoculum size in controlled human malaria infection studies. Malar J.

[7] Arkush KD, Miller MA, Leutenegger CM, Gardner IA, Packham AE, Heckeroth AR (2003). Molecular and bioassay-based detection of *Toxoplasma*
*gondii* oocyst uptake by mussels (*Mytilus*
*galloprovincialis*). Int J Parasitol.

[8] Miller WA, Gardner IA, Atwill ER, Leutenegger CM, Miller MA, Hedrick RP (2006). Evaluation of methods for improved detection of *Cryptosporidium* spp. in mussels (*Mytilus*
*californianus*). J Microbiol Methods..

[9] Knopp S, Salim N, Schindler T, Voules DAK, Rothen J, Lweno O (2014). Diagnostic accuracy of Kato-Katz, FLOTAC, Baermann, and PCR methods for the detection of light-intensity hookworm and *Strongyloides*
*stercoralis* infections in Tanzania. Am J Trop Med Hyg.

[10] Kolapo TU, Bouchard É, Wu J, Bassil M, Revell S, Wagner B (2021). Copro-polymerase chain reaction has higher sensitivity compared to centrifugal fecal flotation in the diagnosis of taeniid cestodes, especially *Echinococcus* spp, in canids. Vet Parasitol.

[11] Llewellyn S, Inpankaew T, Nery SV, Gray DJ, Verweij JJ, Clements AC, Gomes SJ, Traub R, McCarthy JS (2016). Application of a multiplex quantitative PCR to assess prevalence and intensity of intestinal parasite infections in a controlled clinical trial. PLoS Negl Trop Dis.

[12] Papaiakovou M, Pilotte N, Baumer B, Grant J, Asbjornsdottir K, Schaer F, Hu Y, Aroian R, Walson J, Williams SA (2018). A comparative analysis of preservation techniques for the optimal molecular detection of hookworm DNA in a human fecal specimen. PLoS Negl Trop Dis.

[13] Leutenegger CM, Lozoya CE, Tereski J, Savard C, Ogeer J, Lallier R (2023). Emergence of *Ancylostoma*
*caninum* parasites with the benzimidazole resistance F167Y polymorphism in the US dog population. Int J Parasitol Drugs Drug Resist.

[14] Covacin C, Aucoin DP, Elliot A, Thompson RCA (2011). Genotypic characterisation of *Giardia* from domestic dogs in the USA. Vet Parasitol.

[15] Evason MD, Jenkins EJ, Kolapo TU, Mitchell KD, Leutenegger CM, Peregrine AS (2023). Novel molecular diagnostic (PCR) diagnosis and outcome of intestinal * Echinococcus *
* multilocularis * in a dog from western Canada. J Am Vet Med Assoc.

[16] Venkatesan A, Jimenez Castro PD, Morosetti A, Horvath H, Chen R, Redman E (2023). Molecular evidence of widespread benzimidazole drug resistance in *Ancylostoma*
*caninum* from domestic dogs throughout the USA and discovery of a novel β-tubulin benzimidazole resistance mutation. PLoS Pathog.

[17] Massetti L, Colella V, Zendejas PA, Ng-Nguyen D, Harriott L, Marwedel L, Wiethoelter A, Traub RJ (2020). High-throughput multiplex qPCRs for the surveillance of zoonotic species of canine hookworms. PLoS Negl Trop Dis.

[18] Leutenegger CM, Klein D, Hofmann-Lehmann R, Mislin C, Hummel U, Böni J (1999). Rapid FIV provirus quantification by PCR using the TaqMan® fluorogenic real time detection system. J Virol Methods.

[19] Windsor RC, Johnson LR, Sykes JE, Drazenovich TL, Leutenegger CM, De Cock HE (2006). Molecular detection of microbes in nasal tissue of dogs with idiopathic lymphoplasmacytic rhinitis. J Vet Intern Med.

[20] Guy RA, Xiao C, Horgen PA (2004). Real-time PCR assay for detection and genotype differentiation of *Giardia*
*lamblia* in stool specimens. J Clin Microbiol.

[21] McHugh ML (2012). Interrater reliability: the kappa statistic. Biochem Med (Zagreb).

[22] Drake J, Sweet S, Baxendale K, Hegarty E, Horr S, Friis H (2022). Detection of *Giardia* and helminths in Western Europe at local K9 (canine) sites (DOGWALKS Study). Parasit Vectors.

[23] Zendejas-Heredia PA, Colella V, Hii SF, Traub RJ (2021). Comparison of the egg recovery rates and limit of detection for soil-transmitted helminths using the Kato-Katz thick smear, faecal flotation and quantitative real-time PCR in human stool. PLoS Negl Trop Dis.

[24] Miswan N, Singham GV, Othman N (2022). Advantages and limitations of microscopy and molecular detections for diagnosis of soil-transmitted helminths: an overview. Helminthol.

[25] Keller L, Patel C, Welsche S, Schindler T, Hürlimann E, Keiser J (2020). Performance of the Kato-Katz method and real time polymerase chain reaction for the diagnosis of soil-transmitted helminthiasis in the framework of a randomised controlled trial: treatment efficacy and day-to-day variation. Parasit Vectors.

[26] Khurana S, Singh S, Mewara A (2021). Diagnostic techniques for soil-transmitted helminths—recent advances. Res Rep Trop Med.

[27] Kotze AC, Gilleard JS, Doyle SR, Prichard RK (2020). Challenges and opportunities for the adoption of molecular diagnostics for anthelmintic resistance. Int J Parasitol Drugs Drug Resist.

[28] Prichard RK, von Samson-Himmelstjerna G, Blackhall WJ, Geary TG (2007). Foreword: towards markers for anthelmintic resistance in helminths of importance in animal and human health. Parasitol.

[29] Adeyemo FE, Singh G, Reddy P, Stenström TA (2018). Methods for the detection of *Cryptosporidium* and *Giardia*: from microscopy to nucleic acid based tools in clinical and environmental regimes. Acta Trop.

[30] Bowman DD, Mika-Grieve M, Grieve RB (1987). Circulating excretory-secretory antigen levels and specific antibody responses in mice infected with *Toxocara*
*canis*. Am J Trop Med Hyg.

[31] Thompson RCA, Ash A (2019). Molecular epidemiology of *Giardia* and *Cryptosporidium* infections—what's new?. Infect Genet Evol.

[32] Gates MC, Nolan TJ (2009). Comparison of passive fecal flotation run by veterinary students to zinc-sulfate centrifugation flotation run in a diagnostic parasitology laboratory. J Parasitol.

[33] Camacho M, Reinhard KJ (2019). Confusing a pollen grain with a parasite egg: an appraisal of “paleoparasitological evidence of pinworm (*Enterobius*
*vermicularis*) infection in a female adolescent residing in ancient Tehran”. Korean J Parasitol.

[34] Nejsum P, Andersen KL, Andersen SD, Thamsborg SM, Tejedor AM (2020). Mebendazole treatment persistently alters the size profile and morphology of *Trichuris*
*trichiura* eggs. Acta Trop.

[35] Labuschagne M, Beugnet F, Rehbein S, Guillot J, Fourie J, Crafford D (2018). Analysis of *Dipylidium*
*caninum* tapeworms from dogs and cats, or their respective fleas—Part 1. Molecular characterization of *Dipylidium*
*caninum*: genetic analysis supporting two distinct species adapted to dogs and cats. Parasite.

[36] Sweet S, Hegarty E, McCrann DJ (2021). A 3-year retrospective analysis of canine intestinal parasites: fecal testing positivity by age, US geographical region and reason for veterinary visit. Parasit. Vectors..

